# Bilingualism, Demographics, and Cognitive Control: A Within-Group Approach

**DOI:** 10.3389/fpsyg.2020.00094

**Published:** 2020-01-29

**Authors:** Zhilong Xie, Shuya Zhou

**Affiliations:** Foreign Languages College, Jiangxi Normal University, Nanchang, China

**Keywords:** bilingualism, demographics, cognitive control, conflict monitoring, inhibition, mental set shifting

## Abstract

Previous studies have suggested a bilingual advantage in cognitive control as a result of the bilinguals’ language experience. However, the results are controversial as there are various factors (language proficiency, SES, culture, and intelligence, etc.) affecting cognitive control. In the current study, after between-group comparisons, we adopted a within-group approach by multiple regressions to investigate whether the performance by 10-to-75-year-old participants (*N* = 91) of tasks measuring inhibition, monitoring, and mental set shifting could be predicted by bilingualism, or demographic factors, or both. The results of multiple stepwise regression analyses showed that L2 proficiency was a significant predictor for conflict monitoring and inhibition, education and age were significant predictors for mental set shifting, and SES was a minor predictor for inhibition. These findings provide evidence that cognitive control is affected by both bilingualism and demographic factors. Future studies are encouraged to further identify the relationship between bilingualism and cognitive control from specific bilingual experience.

## Introduction

Bilingualism is a common phenomenon, which includes both simultaneous bilinguals who learn two languages simultaneously at early age, and sequential bilinguals who learn a second or foreign language after the acquisition of the first language. The relationship between bilingualism and cognition has been a topic of great concern. Previous research shows that bilinguals have advantage over monolingual counterparts in cognitive control (Peal and Lambert, 1962; Bialystok et al., 2004, 2009; Costa et al., 2009; Prior and Gollan, 2011). Cognitive control is a complex mental process that individuals use to manage behaviors and thoughts, which is a composition of multiple dimensions such as inhibition, mental set shifting, working memory updating, attention, and conflict monitoring (Miyake et al., 2000; Bialystok et al., 2009; Green and Abutalebi, 2013; Bialystok, 2017). Later research shows that bilingual advantage can also be reflected in neural physiological studies in that bilinguals preserve higher efficiency in neural networks in terms of the density/volume of gray or white matter compared to monolinguals, which is assumed to delay cognitive aging (Gold et al., 2013; Abutalebi and Clahsen, 2014; Abutalebi et al., 2014; Olsen et al., 2015). The bilingual advantage has been thought to come from inhibitory control training in bilingual language use. It is reported that when bilinguals intend to use the target language, the non-target language is also activated in the brain (Abutalebi and Green, 2007; Kroll et al., 2008; Hoshino and Thierry, 2011), so in order to successfully use the target language, an Inhibitory Control system (Green, 1998) is adopted to focus on the target information and at the same time suppress the non-target information. Thus, in such a long-term language control experience, inhibitory control system becomes more efficient and finally transfers to non-linguistic domain. Research indicates that linguistic control and non-linguistic control activate similar neural networks (Abutalebi and Green, 2007; Wu et al., 2019), which suggests a common or shared neural mechanism for language control and general cognitive control. If language control activates a neural network that is responsible for general cognitive control, it is reasonable to speculate that such language control training may give rise to the enhancement of general cognitive control abilities, i.e., bilingual advantage.

However, there is a contentious debate on the bilingual advantage. Quite a few studies did not find evidence. Hilchey and Klein (2011)’s seminal review compared previous studies on the Simon effect/flanker interference to examine inhibitory control between bilinguals and monolinguals. Their conclusion indicates little or no bilingual advantage among children and young adult groups, whereas it is robust in the middle-aged and old-aged participants and it is more obvious in global RT advantage, suggesting better conflict monitoring, but not inhibitory control.

After their review, more studies have questioned the existence of bilingual advantage. Paap and Greenberg (2013) conducted three studies to compare a large sample of young adult bilinguals (*n* = 122) and monolinguals (*n* = 151) on the bilingual advantage regarding 15 indicators of cognitive control. Multiple tasks including Antisaccade (Study 1), Simon (Studies 1–3), flanker (Study 3), and color-shape switching (Studies 1–3) were administered to measure inhibition, conflict monitoring, and mental set shifting. However, the results showed no bilingual advantage on any indicator measured in the above tasks, and there was no consistency between different measuring tasks.

After Paap and Greenberg’s study, the number of papers challenging the bilingual advantage increased notably. Quite a few scholars have doubted the significance and validity of the current bilingual advantage research and have claimed that bilingual advantages may not exist or are restricted to specific circumstances (Anton et al., 2014; Gathercole et al., 2014; Paap et al., 2015; Sanchez-Azanza et al., 2017; Donnelly et al., 2019). Some meta-analyses reviewed previous studies on bilingual advantage but reported mixed results. Lehtonen et al. (2018) compared bilinguals’ and monolinguals’ performance in six cognitive control dimensions by using effect sizes from 152 previous studies on adults. They concluded that their analyses revealed only a small bilingual advantage in dimensions of inhibition, shifting, and working memory, but no bilingual advantage in monitoring or attention. Furthermore, after correcting estimates for observed publication bias, they found no evidence for a bilingual advantage at all. However, van den Noort et al. (2019)’s findings are contradictory. They searched the Medline, ScienceDirect, Scopus, and ERIC databases for all original data and reviewed studies on bilingualism and cognitive control, with a cut-off date of October 31, 2018. After the meta-analyses, they found that the majority, 54.3%, reported beneficial effects of bilingualism on cognitive control tasks, 28.3% mixed results, and 17.4% against its existence. These inconsistent findings have led to some doubts about the veracity of bilingual advantage and thus the current debate in the field (Antoniou, 2019; de Bruin, 2019; Paap, 2019).

How to interpret the contradictory findings? A range of factors are thought to account for the inconsistent results. Firstly, some scholars claimed that the field suffers from publication bias, which suggested that studies supporting the bilingual advantage hypothesis were most likely to be published, whereas the ones challenging it were less likely to be published (de Bruin et al., 2015).

Secondly, cognitive control has been reported to be modulated by participant relevant variables, including age (Tao et al., 2011), socio-economic status – SES (Morton and Harper, 2007), culture (Yang et al., 2011), intelligence (Xie and Pisano, 2019), and immigration status (Kousaie and Phillips, 2012). Specifically, young adult participants have been reported to perform much better than children and older adults because they are in the peak development of cognitive control, and bilingual advantages are more robust among older adult bilinguals as their cognitive control is in decline (Bialystok et al., 2009). Participants with higher SES tend to have better cognitive control abilities (Calvo and Bialystok, 2014), and better cognitive control is linked to higher scores on intelligence tests (Xie and Pisano, 2019).

Thirdly, benefits from bilingualism are inconsistent because individuals vary in the number and types of experiences they have that promote superior cognitive control. It has been argued that the variability in the language context of bilingual speakers (or variability of bilingual experience) may modulate cognitive control in different contexts (Green and Abutalebi, 2013). For example, bilinguals who have more practice of switching languages may be superior in mental set shifting compared to monolinguals or bilinguals who do not have such practice (Prior and Gollan, 2011; Yudes et al., 2011). Bilinguals with higher L2 proficiency or more practice of L2 speaking training may have better ability in conflict monitoring (Xie and Dong, 2017; Xie and Pisano, 2019). Bilinguals may also show better conflict monitoring in high demanding version of the flanker task (Costa et al., 2009). To sum up, it has been suggested that the mixed results of bilingual advantage could have come from multiple variables: particularly, the varying demographic features of the bilingual participants, and the individual differences of bilingual experience.

Although previous studies have assessed different factors affecting cognitive control, most of them relied on quasi-experimental designs where bilinguals were compared to monolinguals (or compared to different bilingual groups). Such designs are fruitful in providing evidence of bilingual advantage, but this kind of design more or less entails the randomization of participants into the different groups. Thus, it is difficult to exclude the role of these confounding factors, which may co-vary with relevant variables. One way to avoid such between-group confounding effects is to conduct longitudinal research. For example, Crivello et al. (2016) examined whether growth in bilingualism (indicated by increased number of translation equivalents) improves cognitive control over a period of 7-month time. Bilingual children were measured on expressive vocabulary and translation equivalents and on a battery of cognitive control tasks (conflict, delay, and working memory) when they were 24 and 31 months of age. It was found that within the bilingual participants, increase of the number of translation equivalents, rather than vocabulary growth, predicted the better performance on conflict tasks but not on delay tasks. This within-group design confirmed the relation between bilingualism and cognitive control. Another way to avoid bilingual and monolingual comparison consists in examining the impact of the bilingual experiences within bilingual participants. For example, Soveri et al. (2011) used multiple regression to study whether the performance of 30- to 75-year-old Finnish–Swedish bilinguals (*N* = 38) on cognitive control tasks (inhibition, updating, and mental set shifting) could be predicted by language switching experience, L2 age of acquisition, or by the language proficiency. The results showed that a higher rate of everyday language switches was related to a smaller mixing cost in errors, which reflected top-down management of competing task sets, resembling the bilingual situation where decision of which language to use has to be made in certain language context. In a more recent example, DeLuca et al. (2019b) investigated whether differences in bilingual experiences could confer systematic brain adaptations among 65 healthy right-handed bilinguals (mean age: 31.7, range: 18–52). Correlation and regression models revealed that L2 AoA was found to significantly predict expansions in the left nucleus accumbens and the bilateral thalamus. Specifically, length of L2 immersion significantly predicted significant adaptations in posterior sections of the right caudate nucleus; years of active L2 use predicted an expansion in the left nucleus accumbens, and active L2 immersion predicted both an expansion and contractions in the right caudate nucleus. Briefly, specific experience-based factors related to bilingualism (such as extent and duration of active L2 use) predicted specific adaptations in the brain. This study sets an example of investigating how different dimensions of bilingualism may affect brain structures and functions within bilinguals (DeLuca et al., 2019a).

Following this line of research, the current study intends to investigate the relationship between bilingualism and cognitive control by introducing a similar within-group approach. We employ multiple regressions to investigate whether participants’ performance on tasks measuring three aspects of cognitive control (inhibition, conflict monitoring, and mental set shifting) could be predicted by either bilingualism itself or participant relevant factors (such as SES, age, and IQ), or both. We hypothesize that both factors may be significant predictors of cognitive control but in different dimensions.

## Materials and Methods

### Participants

The current study included 91 healthy, right-handed English, Chinese monolinguals and Chinese-English bilinguals between 10 and 75 years of age (*M* = 26.45). The English monolinguals (*n* = 26, limited L2 s: Spanish, Chinese, and French) were participants from New York City and the suburban area. The Chinese monolinguals (*n* = 31, limited L2: English) and the Chinese-English bilinguals (*n* = 34) were participants from the Jiangxi Province of China and Jiangxi Normal University, respectively. All participants voluntarily participated in the study and gave informed consent, and their rights were protected in accord with the ethical standards of the Academic Committee of Jiangxi Normal University.

On the average, they were quite highly educated (*M* = 14.02 years). All the monolinguals had very limited L2 proficiency. The English monolinguals had a mean of 4.62 (out of 40) for L2 proficiency. The Chinese monolinguals had a mean of 7.06 for L2 proficiency. Therefore, although they were considered as monolinguals in the current study, they could also be classified as bilinguals with a relatively low L2 proficiency. All the bilinguals were Chinese native speakers who formally learnt English as a foreign language at around age 10 in school (*Mean* of L2 proficiency = 23.38). In order to obtain participants’ demographic information and their language proficiency, we adopted the language experience and proficiency questionnaire (Marian et al., 2007). Such questionnaires are widely used in bilingual research, and are significantly correlated with objective measures of language proficiency (Marian et al., 2007; Prior and Gollan, 2011). In the language proficiency questionnaire, language skills were composed of listening, speaking, reading, and writing, respectively in L1 and L2 on a scale from 0 to 10, where 0 corresponded to no skills in that particular language and 10 to skills at a native level. There was a significant difference between their L1 and L2 language proficiency (33.70 vs. 12.46), *t* = 19.598, *p* < 0.001, and all the groups differed in L2 proficiency (*p*s < 0.05, see Table 1).

**TABLE 1:** Demographic scores of all participants (*N* = 91).

	English	Chinese	Chinese-English	Total
	Monolinguals	Monolinguals	Bilinguals	M (SD)
	(*n* = 26)	(*n* = 31)	(*n* = 34)	(*n* = 91)
Age (years)	37.65^b^ (19.21)	21.55^a^ (3.56)	22.35^a^ (1.86)	26.45 (12.60)
Education	14.21^b^ (3.95)	11.29^a^ (2.51)	16.35^c^ (1.86)	14.02 (3.51)
(years)				
SES (1–7)	3.69^b^ (1.34)	2.68^a^ (1.58)	2.69^a^ (1.58)	2.97 (1.57)
IQ (0–72)	63.58 (5.16)	65.00 (5.59)	64.79 (5.34)	64.52 (5.35)
L1 Proficiency*	34.31 (5.68)	31.62 (2.62)	35.15 (1.78)	33.70 (3.85)
L2 Proficiency*	4.62^a^ (5.85)	7.06^b^ (2.87)	23.38^c^ (4.37)	12.46 (9.59)

*Total scores of language proficiency = 40 (listening + speaking + reading + writing). Means in the same row with different superscript letters differ from each other significantly at *p* < 0.01.

Furthermore, we adopted *Ravens Matrices* (Raven et al., 1977; Li, 1989) to measure participants’ IQ and used participants’ parental education as proxy of SES^1^ (Wermelinger et al., 2017). The results showed no significant group differences in IQ (*F* < 1, *p* = 0.569), but significant group differences in SES. English monolinguals had a significant higher level of SES compared to the other two similar groups (*p* = 0.020). Moreover, the three groups also differed in age, with the English monolinguals much older than the other two (see Table 1).

### Cognitive Control Tasks

In the current study, we employed two cognitive control tasks to measure three dimensions: inhibition, conflict monitoring, and mental set shifting.

### The Flanker Task

The flanker task (Eriksen and Eriksen, 1974) has been widely used to measure cognitive control, of which inhibition and conflict monitoring are two main components. Inhibition indicates the ability to suppress responses that are inappropriate in a given situation (Luk et al., 2010; Paap and Greenberg, 2013), indicated by the RT differences between incongruent trials and congruent trials, which is a long established measure in the study of bilingual advantage. Conflict monitoring indicates the ability to monitor one’s performance or internal state, or monitor the context and evaluate whether conflict resolution processes should be involved when the target information is presented (Costa et al., 2009; Paap and Greenberg, 2013). This was first proposed by Bialystok et al. (2004), who suggested that the bilingual advantage (of faster speed) both for congruent trials and incongruent trials on the Simon task may reflect “the ability to manage attention to a complex set of rapidly changing task demands”(p. 292). Costa et al. (2009) adopted a flanker task and put forward that “the bilingual advantage in overall RTs may reveal the better ability of bilinguals to handle tasks that involve mixing trials of different types: bilinguals would be more efficient at going back and forth between trials that require implementing conflict resolution and those that are free of conflict” (p. 136). Managing two languages may enhance conflict monitoring because “the bilinguals need to continuously monitor the appropriate language for each communicative interaction. That is, proper communication in bilingual settings involves the monitoring of the language to be used depending on the interlocutor(s) language knowledge” (p. 136). The indicator for conflict monitoring is sometimes the RT difference between trials in a pure block and the congruent trials in a block where congruent and incongruent trials are mixed, sometimes RT on the congruent trials, or RT on both the congruent and incongruent trials (Bialystok et al., 2004; Costa et al., 2009; Paap and Greenberg, 2013). In the current study, we use the RTs on each condition as the indicator of conflict monitoring (as in Dong and Xie, 2014; Xie and Dong, 2017).

In the current flanker task, there were three conditions: congruent, incongruent, and neutral. In the congruent condition, the target chevron (in red color) was flanked by black chevrons pointing to the same direction, thus creating facilitation. In the incongruent condition, the target chevron was flanked by black chevrons pointing to the opposite direction, thus creating conflict. In the neutral condition, the target chevron was flanked by black diamond symbols that share no shape similarity, thus creating no conflict or facilitation.

The flanker task, adapted from previous studies (Luk et al., 2010; Xie and Dong, 2017), was computerized via E-prime 2.0. The task was composed of a practice block with feedback (smiling face for correct, frowning face for incorrect) and a formal experimental block without feedback. In each trial, there was a fixation stimulus of “ + ” for 250 ms. Then each condition of the stimulus appeared randomly for 2000 ms. After that, participants were required to press the designed button corresponding to the direction of each target red chevron. A new trial would start again after the participant’s response or 2000 ms. One thing to note is that in the task, each participant would not begin the formal experiment until he or she performed with an accuracy rate above 80% in the practice block, which was to ensure focused attention on the task. There were altogether 108 trials in the experimental block.

### The Wisconsin Card Sorting Test (WCST)

One of the most widely used tasks to measure mental set shifting is the Wisconsin Card Sorting Test (Barceló and Knight, 2002; Moriguchi and Hiraki, 2009; Nyhus and Barcelo, 2009). Mental set shifting is the ability to shift back and forth between multiple tasks, operations, or mental sets (Monsell, 1996; Miyake et al., 2000). In this test, based on four stimulus cards that included one red triangle, two green stars, three yellow crosses, and four blue circles, participants were required to classify the response card, which was a constellation of these numbers, colors, and shapes. Participants were presented with feedback of correctness after each response, and based on the feedback they could deduce new implied rules to classify the next trial. What was unknown to the participants was that the implied rule would change after a few trials (varied from 5–9 trials).

The computerized version of WCST programed in E-prime 2.0, which followed previous studies (Yudes et al., 2011; Dong and Xie, 2014; Xie and Pisano, 2019), was composed of 12 practice trials and 128 formal experimental trials. Each trial began with a fixation “ + ” for 1000 ms. After that the four stimulus cards appeared in the upper half of the screen while a response card appeared at the same time in the lower half of the screen. Participants were required as soon as possible to sort out the response card according to any of the stimulus cards by pressing designated buttons. Then a feedback appeared for 1000 ms before the next trial. In performing the task, there was an optional break in the middle.

### Statistical Analyses

To provide a comprehensive result, we first conducted a repeated measures analyses with condition as within-group variable and group as between-group variable, and then we conducted multiple linear step-wise regression analyses separately for each task, with the processing response times (RTs) (conflict monitoring) and the flanker effect (inhibition) in the flanker task, the overall performance of completed categories and errors in the WCST (mental set shifting) as dependent variables, and with L1 proficiency, L2 proficiency, SES, age, education, and IQ as independent variables.

## Results

For the flanker task, we calculated correct response times, excluding errors and correct responses that fell three standard deviations outside the mean time for each subject in each condition, which occupied for 2.52% of the total data. For the WCST, we calculated overall RTs, completed categories, overall errors, and types of errors independently.

### Flanker Task

In the flanker task, as mentioned above two indices were calculated to assess participants’ performances (Bialystok et al., 2004; Costa et al., 2009; Paap and Greenberg, 2013). The first one is flanker effect, which is the response-time difference between incongruent trials and congruent trials. The flanker effect has been considered as an indicator of inhibition. Smaller flanker effect indicates better ability of inhibition (inhibitory control). The second one is response times in each condition, which is considered as an indicator of conflict monitoring, reflecting an ability to monitor a context where conflict occurs or not. Faster RTs indicate better ability of conflict monitoring. Data of all participants’ performances are presented in Table 2.

**TABLE 2:** Flanker task performance across groups (*N* = 91).

	English	Chinese	Chinese-English
	Monolinguals	Monolinguals	Bilinguals
	(*n* = 26)	(*n* = 31)	(*n* = 34)
	*M* (SD)	*M* (SD)	*M* (SD)
Congruent condition	600.4^b^ (118.9)	534.5^a^ (105.4)	522.8^a^ (78.8)
Neutral condition	619.5^b^ (115.6)	553.4^a^ (89.1)	548.5^a^ (91.6)
Incongruent condition	642.3 (112.7)	593.6 (86.8)	591.0 (85.0)
Flanker effect	41.9 (50.5)	59.1 (40.3)	68.2 (49.5)

*Means in the same row with different superscript letters differ from each other significantly at *p* < 0.05.*

Repeated measures analyses with condition as within-group variable and group as between-group variable were conducted to examine whether there were differences across conditions and across groups. Results of within-subjects effects revealed a significant effect of condition, *F*(2,176) = 72.035, *p* < 0.001, η^2^ = 0.450, but no significant condition and group interaction, *F*(4,176) = 1.460, *p* = 0.216, η^2^ = 0.032. Planned comparisons showed that all participants responded more quickly in the congruent condition (548.94 ms) than in the neutral (570.44 ms), *F*(1,90) = 19.665, *p*< 0.001, η^2^ = 0.179, and the incongruent conditions (606.53 ms), *F*(1,90) = 133.277, *p* < 0.001, η^2^ = 0.597. Participants also responded more quickly in the neutral condition (570.44 ms) than in the incongruent condition (606.53 ms), *F*(1,90) = 68.389, *p*< 0.001, η*^2^* = 0.432.

**FIGURE 1:**
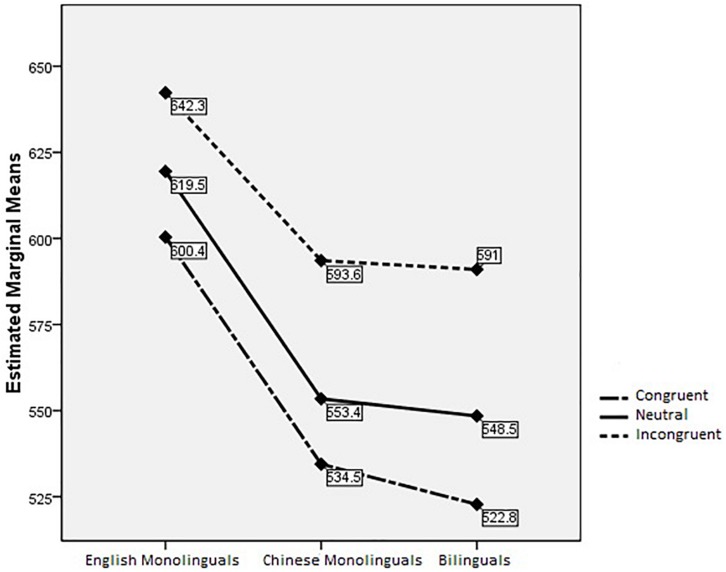
Flanker task.1pc performance across groups and conditions.

Results of between-subjects effects showed that there were significant differences between groups, *F*(2,88) = 4.262, *p* = 0.017,η^2^ = 0.088. ANOVA analyses results showed significant group differences on the congruent condition (*p* = 0.010) and the neutral condition (*p* = 0.013) but not on the incongruent condition or on the flanker effect (*p*s > 0.05). Results of *post hoc* bonferroni multiple comparisons showed significant differences between the English monolinguals and the Chinese monolinguals on congruent condition and neutral condition (*p* = 0.047, *p* = 0.040, respectively), between the English monolinguals and the Chinese-English bilinguals on congruent and neutral conditions (*p* = 0.012, *p* = 0.020, respectively), but not between the Chinese monolinguals and the Chinese-English bilinguals (*p*s > 0.05) (see Figure 1).

However, besides different L2 proficiency across groups, demographic variables such as age, SES, and education were also different across the three groups (*p*s < 0.05, see Table 1), so we could not tell what was the real factor(s) for the differences on the flanker task performance. Apparently, the English monolinguals were much older (*M* = 37.65) than the other groups (*M* = 21.55, 22.35, respectively), although they had higher SES (*M* = 3.69) compared to the other two groups (*M* = 2.68, 2.69, respectively). These results may be caused by confounding factors and thus need further analyses.

Therefore, in order to find out what variables may predict the performance of the flanker task, we conducted step-wise multiple regression analyses with RTs of each condition and the flanker effect as dependent variables, with L1 proficiency, L2 proficiency, SES, age, education, and IQ as independent variables. The results of multiple regressions on the flanker congruent condition showed that only L2 proficiency remained in the model in predicting the RTs of congruent condition, *R* = 0.297, *R*^2^ = 0.088, adjusted *R*^2^ = 0.078, *F*(1,89) = 8.591, *p* = 0.004. Other variables such as age, education, SES, IQ, and L1 proficiency were excluded from the model (Table 3).

**TABLE 3:** Results of the multiple regressions for the flanker task performance.

Predictors	UC*		SC*	Dependent variable	*t*	Sig.
	*B*	Std. error	Beta			
L2 proficiency	−3.244	1.107	−0.297	Congruent RTs	−2.931	0.004
L2 proficiency	−2.662	1.091	−0.250	Neutral RTs	−2.440	0.017
Education	−8.169	2.768	−0.299	Incongruent RTs	−2.951	0.004
SES	−6.734	3.086	−0.221	Flanker effect	−2.182	0.032
L2 proficiency	−1.033	0.503	−0.208		−2.052	0.043

*UC= unstandardized coefficients; SC= standardized coefficients.

The results of multiple regressions on the flanker neutral condition similarly showed that only L2 proficiency significantly predicted the RTs of neutral condition, *R* = 0.250, *R*^2^ = 0.063, adjusted *R*^2^ = 0.052, *F*(1,89) = 5.955, *p* = 0.017. Other variables such as age, education, SES, IQ, and L1 proficiency were excluded from the model (Table 3).

However, the results of multiple regressions on the flanker incongruent condition showed that only education significantly predicted the RTs of incongruent condition, *R* = 0.299, *R*^2^ = 0.089, adjusted *R*^2^ = 0.079, *F*(1,89) = 8.708, *p* = 0.004. Other variables such as age, SES, IQ, L1 proficiency, and L2 proficiency were excluded from the model (Table 3).

In addition, we conducted multiple regression analyses on the flanker effect, which is an indicator of inhibition. The results of multiple regressions on the flanker effect showed that both SES and L2 proficiency significantly predicted the flanker effect, *R* = 0.317, *R*^2^ = 0.100, adjusted *R*^2^ = 0.080, *F*(1,89) = 4.906, *p* = 0.010. Other variables such as age, IQ, and L1 proficiency were excluded from the model (Table 3).

To summarize, the overall results of the multiple regression analyses of the flanker task showed that L2 proficiency predicted the congruent condition RTs and the neutral condition RTs, education predicted the incongruent condition RTs, and SES and L2 proficiency predicted the flanker effect. As mentioned above, RTs reflect the ability of conflict monitoring and flanker effect reflects the ability of inhibition, so we can generally conclude that L2 proficiency was a major predictor of conflict monitoring whereas education was a minor. Furthermore, both SES and L2 proficiency significantly predicted inhibition.

### WCST

In the WCST, we calculated five indices to reflect mental set shifting. The first one is overall response times, which reflected participants’ speed during the task. The second one is the total number of correct categories participants completed. The third one is the total number of errors participants made. The fourth one is the total number of perseverative errors that participants failed to change to a correct mental rule after receiving negative feedback. The fifth one is the total number of previous category errors that participants continued in sorting cards according to the previous category dimension despite the negative feedback. Data of all groups’ performances are presented in Table 4.

**TABLE 4:** WCST performance across groups (*N* = 91).

	English	Chinese	Chinese-English
	Monolinguals	monolinguals	bilinguals
	(*n* = 26)	(*n* = 31)	(*n* = 34)
	*M* (SD)	*M* (SD)	*M* (SD)
WCST RTs	1693.7 (602.5)	1383.7 (549.7)	1549.1 (521.0)
Completed category	8.9^b^ (3.7)	5.9^a^ (2.7)	9.5^b^ (3.4)
Overall errors	46.8^a^ (17.2)	73.2^c^ (13.4)	56.3^b^ (13.5)
Perseverative errors	25.2^a^ (17.7)	54.4^c^ (15.4)	35.9^b^ (13.8)
Previous category errors	12.7^a^ (12.9)	40.5^b^ (17.6)	18.0^a^ (9.7)

*Means in the same row with different superscript letters differ from each other significantly at *p* < 0.05.*

ANOVA analyses results showed that there were no RT differences across the groups (*p* > 0.05). However, there were significant group differences on completed category *F*(2,90) = 10.975, *p* < 0.001, overall errors *F*(2,90) = 24.149, *p* < 0.001, perseverative errors *F*(2,90) = 26.027, *p* < 0.001, and previous category errors *F*(2,90) = 34.287, *p* < 0.001. *Post hoc* bonferroni multiple comparisons showed that both the English monolinguals and the Chinese-English bilinguals completed more categories than the Chinese monolinguals (*p* = 0.002, *p* < 0.001, respectively). The English monolinguals produced fewer overall errors and perseverative errors than the Chinese-English bilinguals (*p* = 0.046, *p* = 0.029), and the Chinese-English bilinguals produced fewer errors than the Chinese monolinguals (*p*s < 0.001). Finally, the English monolinguals and the Chinese-English bilinguals produced similar previous category errors but both were better than the Chinese monolinguals (*p*s < 0.001). These mixed results are actually complicated. As we have mentioned earlier in the flanker task performance, the three groups differed in L2 proficiency and in demographic factors such as age, SES, and education, so it is difficult to speculate which factor or a combination of the factors might have contributed to the differences.

Therefore, similar to the flanker task, in order to find out what variables contributed to the performance of the WCST task, we conducted step-wise multiple regression analyses with each index as a dependent variable, whereas L1 proficiency, L2 proficiency, SES, age, education, and IQ as independent variables. The results of multiple regressions on the overall RTs showed that age was the only predictor for the overall RTs of the WCST, *R* = 0.357, *R*^2^ = 0.127, adjusted *R*^2^ = 0.117, *F*(1,89) = 12.965, *p* = 0.001 (see Table 5). Other variables such as L2 proficiency, education, SES, IQ, and L1 proficiency were excluded from the model (*p*s > 0.05).

**TABLE 5:** Results of the multiple regressions for WCST.

Predictors	UC*		SC*	Dependent variable	t	Sig.
	*B*	Std. error	Beta			
Age	16.050	4.457	0.357	RTs	3.601	0.001
Education	0.410	0.101	0.396	Completed category	4.068	0.000
Education	−1.455	0.513	−0.284	Overall errors	−2.839	0.006
Age	−0.397	0.143	−0.278		−2.784	0.007
Education	−1.617	0.551	−0.314	Perseverative errors	−3.163	0.002
Age	−0.426	0.153	−0.273		−2.751	0.007
Education	−0.378	0.508	−0.357	Previous category errors	−3.859	0.000
Age	−0.227	0.141	−0.232		−2.313	0.023

*UC, unstandardized coefficients; SC, standardized coefficients.

The results of multiple regressions on the number of completed categories showed that education was the only factor that remained in the model in predicting the completed categories of the WCST, *R* = 0.396, *R*^2^ = 0.157, adjusted *R*^2^ = 0.147, *F*(1,89) = 16.548, *p* < 0.001 (see Table 5). Other variables such as L2 proficiency, age, SES, IQ, and L1 proficiency were excluded from the model (*p*s > 0.05).

The results of multiple regressions on overall errors, perseverative errors, and previous category errors showed that both education and age were significant predictors (see Table 5): overall errors, *R* = 0.456, *R*^2^ = 0.207, adjusted *R*^2^ = 0.189, *F*(2,88) = 11.520, *p* < 0.001; the perseverative errors, *R* = 0.477, *R*^2^ = 0.227, adjusted *R*^2^ = 0.210, *F*(2,88) = 12.808, *p* < 0.001; the previous category errors, *R* = 0.498, *R*^2^ = 0.248, adjusted *R*^2^ = 0.231, *F*(2,88) = 14.370, *p* < 0.001. Other variables such as L2 proficiency, SES, IQ, and L1 proficiency were excluded from the model (*p*s > 0.05).

To summarize, the overall results of the multiple regression analyses of the WCST showed that age and education mainly predicted the overall performance of the task. There are subtle differences, though, in that age was the only predictor for the RTs, education was the only predictor for the completed categories, whereas both education and age significantly predicted the overall errors and different types of errors. Therefore, in all, we could presumably conclude that age and education are the main factors for mental set shifting.

## Discussion

Given the controversial results of bilingual advantage in cognitive control, the current study set out to explore this issue with a within-group approach where the relationship between multiple factors including bilingualism and cognitive control was investigated. In a sample of 91 participants including English monolinguals, Chinese monolinguals, and Chinese-English bilinguals, we found that L2 proficiency was a significant predictor for cognitive control, particularly in conflict monitoring and inhibition but not in mental set shifting. Broadly speaking, this result provides at least partial support for the hypothesis that the bilingual advantage is related to bilingual experience that requires cognitive control (Green, 1998; Abutalebi and Green, 2007; Bialystok et al., 2009; Costa et al., 2009; Hartanto and Yang, 2019).

The results that bilingualism (indicated by L2 proficiency) is related to inhibition and conflict monitoring (as tested by the flanker task) are consistent with some previous studies. For example, Bialystok and Martin (2004) showed that bilingual children outperformed monolinguals in dimensional card change sorting task. This better inhibitory control reflects the ability of ignoring perceptual information. In another study Bialystok et al. (2008) used a spatial Stroop task to compare the different performances between elderly bilinguals and monolinguals. They demonstrated that bilinguals outperformed monolinguals on the interference effect (inhibition). According to Hilchey and Klein (2011), the bilingual advantage on inhibition is sporadic. However, the result of the current study showed that bilingualism (L2 proficiency) is indeed a significant predictor of inhibition. The relation between bilingualism and conflict monitoring is robust in the current study. L2 proficiency significantly predicted the flanker task performance in both congruent and neutral trials (but no effect for incongruent trials). This finding is partly consistent with a few studies. For example, in Bialystok et al. (2004), bilinguals showed faster response times (monitoring) compared to monolinguals, both in congruent trials and incongruent trials of a Simon task. In Emmorey et al. (2008), a flanker task was adopted to compare the performances between middle-aged bilinguals and monolinguals that were matched on SES. Their results showed that the bilingual participants responded more rapidly on congruent trials and incongruent trials than did the monolinguals, which is partially in conformity with the current finding. Similarly, Costa et al. (2009) examined whether there were different performances between young adult monolinguals and bilinguals in the flanker task under different cognitive demands, and they found that the bilinguals were faster in performing the task when it is under high cognitive demand (by varying the percentage of congruent and incongruent trials). In a more recent study by Xie (2018), it was shown that among the three bilingual groups varying in L2 proficiency, the high L2 proficiency bilingual group performed faster than the low L2 proficiency bilingual group in congruent, neutral, and incongruent trials, which further proves that bilingualism is significantly related to response times, which presumably reflects conflict monitoring.

However, the regression analyses results of the current study showed no relation between bilingualism and mental set shifting. This result may be related to the fact that our participants did not have enough language switching experience. For the monolinguals in our study, they surely did not have such language switching experiences. Even for the Chinese-English bilinguals, they had few opportunities of speaking English. In mainland China, English is taken as a foreign language, so students usually do not have the environment and necessity of speaking English in their daily life, let alone the experience of language switching. Costa et al. (2009) suggested that bilinguals who do not have switching experience may show an advantage in conflict monitoring. On the contrary, studies show that even short-term language switching training can transfer to non-linguistic domain for the mental set shifting among bilinguals (Timmer et al., 2019). This assumed relation between language switching and mental set shifting has support from neuro-imaging studies. For example, studies have shown that different neural networks are activated when different dimensions of cognitive control are required in language use. It is shown that the middle frontal gyrus and left parietal lobe are more active when bilinguals switch between languages or non-linguistic tasks (Sun et al., 2019). This experience-dependent neural effects are in fact consistent with a newly proposed theory – neuroemergentism, which states that the effects of bilingualism on cognitive control is a combination of much smaller systems that are sensitive to age, age of acquisition, proficiency or performance level, specific language experience, and individual difference. The effects are developmental, dynamic, and non-linear, and are likely the products of different forms of bilingual experiences. Thus it is suggested that future research of the bilingual advantage should not center on whether language experience affects cognitive control or not, but on how language experience at different points in life dynamically affects cognitive control (Li et al., 2014; Hernandez et al., 2018, 2019).

Moreover, the current study found that some demographic factors were significantly associated with cognitive control in some dimensions. Specifically, age and education were associated with mental set shifting, whereas SES associated with inhibition, which conflicts with some recent studies in that SES is related to conflict monitoring (reflected by response times) (e.g., Naeem et al., 2018; Xie and Pisano, 2019). Nevertheless, these results are generally in line with the findings that higher education and SES are correlated positively with cognitive control, whereas age is positively correlated with cognitive control for children and young adult participants but negatively correlated with cognitive control for older adults (Valian, 2015; van den Noort et al., 2019; Xie and Pisano, 2019). In the current study, however, we did not find association between cognitive control and intelligence. In Xie and Pisano (2019), intelligence was reported a significant predictor of congruent trials in the flanker task. In Woumans et al. (2016), a longitudinal study was carried out to examine whether immersion bilingual schooling could improve cognitive control. Their results found that the children attending bilingual kindergarten improved not only in cognitive control but also in intelligence. All these results provide evidence that demographic factors do really affect cognitive control, although why and how demographic factors (such as age, education and SES) are related to one dimension but not other remains unknown, which calls for future research to identify how each demographic factor is related to specific dimension of cognitive control under what specific bilingual context.

## Conclusion

In the current study, we used a within-group approach to investigate multiple factors that may potentially affect cognitive control. The results showed that both bilingualism and demographics contributed to cognitive control. Our study presents a complementary methodological approach that will hopefully shed more light on the important issue of the bilingual advantage. It is without doubt that the measurement of the various aspects of bilingual experience, the multiple factors in demographics, and the identification of various dimensions of cognitive control are to be further clarified in future studies.

## Data Availability Statement

The datasets generated for this study are available on request to the corresponding author.

## Ethics Statement

The studies involving human participants were reviewed and approved by the Academic Committee of Jiangxi Normal University. Written informed consent to participate in this study was provided by the participants’ legal guardian/next of kin.

## Author Contributions

All authors listed have made a substantial, direct and intellectual contribution to the work, and approved it for publication.

## Conflict of Interest

The authors declare that the research was conducted in the absence of any commercial or financial relationships that could be construed as a potential conflict of interest.

## Footnotes

1 We recognize that a more objective indicator of SES should include family income, but because our participants did not provide income data and the Chinese-English bilinguals were still college students without income from any profession, we followed Wermelinger et al. (2017) and used all participants’ parental education as an approximate indicator of SES.
